# Supplementary material for: Transcriptomic Leaf Profiling Reveals Differential Responses of the Two Most Traded Coffee Species to Elevated [CO2]
Source: Int J Mol Sci. 2020 Dec 3;21(23):9211. doi: 10.3390/ijms21239211 (PMC7730880; doi:10.3390/ijms21239211)
Supplement: Supplementary file 1 [file ijms-21-09211-s001.zip › Figure S2.pdf]

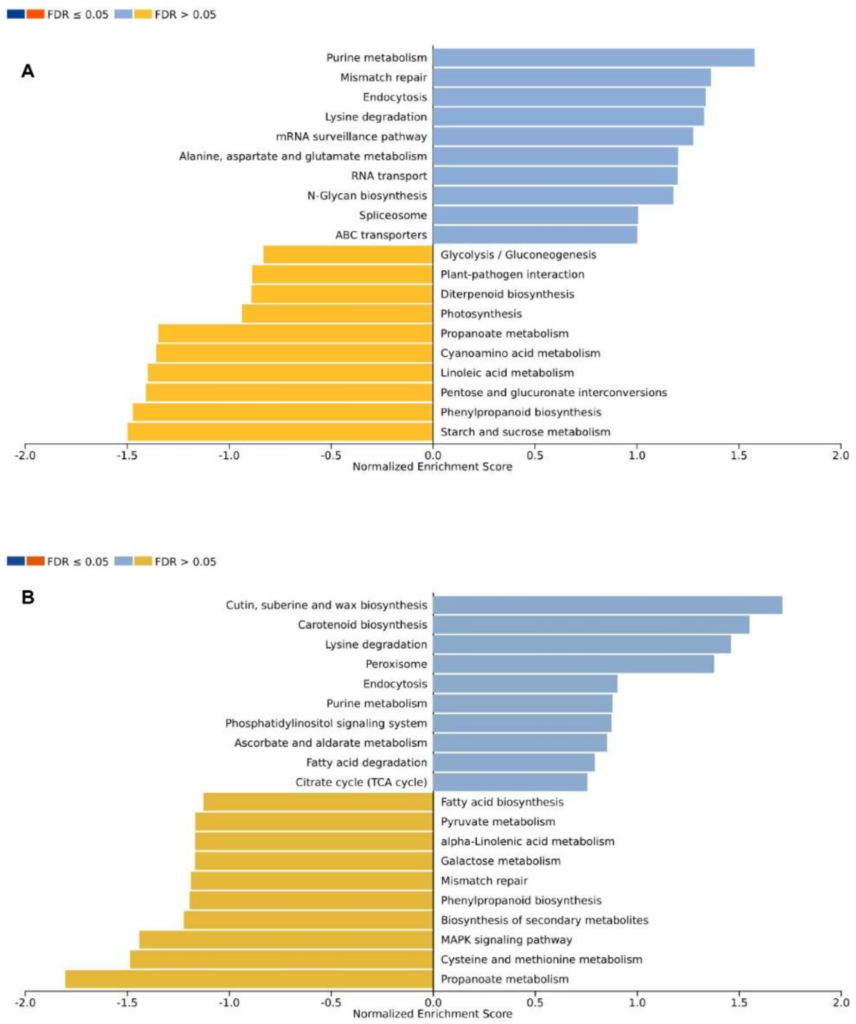

Figure A2. Enriched KEGG metabolic pathways found among DEGs in CL153 vs. Icatu at aCO_2 (A) and eCO_2 (B) showing the highest (blue) and lowest (yellow) normalized enrichment scores. Dark colors indicate statistically significant KEGG metabolic pathways under FDR < 0.05.
