# Supplementary material for: Transcriptomic Leaf Profiling Reveals Differential Responses of the Two Most Traded Coffee Species to Elevated [CO2]
Source: Int J Mol Sci. 2020 Dec 3;21(23):9211. doi: 10.3390/ijms21239211 (PMC7730880; doi:10.3390/ijms21239211)
Supplement: Supplementary file 1 [file ijms-21-09211-s001.zip › Table S1.docx]

**Table A1.** Genome mapping showing the alignment and reads counting results of the transcriptome of ICATU and CL153 against the genome of *Coffee canephora*. A, B, C correspond to the individual biological replicates. RAW READS: number of reads obtained after sequencing. CLEAN READS: number of reads passing the Illumina quality filters and downstream filters. % CLEAN: percentage of reads passing filters compared to the number of raw reads. UNIQUE: number of reads aligned to a unique position. % UNIQUE: proportion of reads aligned to a unique position compared to the number of clean reads. MULTIPLE MAP: number of reads aligned to exons of several overlapping genes. % MULTIPLE MAP: proportion of reads aligned to exons of several overlapping genes compared to the number of clean reads. % UNMAPPED: proportion of non-aligning reads compared to the number of clean reads.

| **GENOTYPES** | **[CO_2_]**  **(µL L^-1^)** | **REPLICATES** | **RAW**  **READS** | **CLEAN**  **READS** | **%**  **CLEAN** | **UNIQUE** | **%**  **UNIQUE** | **MULTIPLE**  **MAP** | **% MULTIPLE**  **MAP** | **%**  **UNMAPPED** |
| --- | --- | --- | --- | --- | --- | --- | --- | --- | --- | --- |
| **ICATU** | 380 | 1A | 28702752 | 26442162 | 92.1 | 23198363 | 87.7 | 2397063 | 9.10 | 3.2 |
|  |  | 1B | 28603251 | 26372236 | 92.2 | 22181864 | 84.1 | 3371170 | 12.7 | 3.1 |
|  |  | 1C | 27795986 | 25107797 | 90.3 | 18468518 | 73.5 | 5832560 | 23.2 | 3.2 |
|  |  | Average | 28367329 | 25974065 | 91.5 | 21282915 | 81.8 | 3866931 | 15.0 | 3.2 |
|  | 700 | 3A | 30895839 | 29009249 | 93.8 | 24211896 | 83.4 | 3872399 | 13.3 | 3.1 |
|  |  | 3B | 25630485 | 23784221 | 92.8 | 18809838 | 79.1 | 4199675 | 17.6 | 3.2 |
|  |  | 3C | 31962251 | 29719153 | 92.9 | 23355288 | 78.5 | 5379009 | 18.1 | 3.3 |
|  |  | Average | 29496191 | 27504207 | 93.2 | 22125674 | 80.3 | 4483694 | 16.3 | 3.2 |
| **CL153** | 380 | 5A | 24532884 | 22853193 | 93.1 | 17904841 | 78.3 | 4300626 | 18.8 | 2.8 |
|  |  | 5B | 28922635 | 26926317 | 93.1 | 20799330 | 77.2 | 5356228 | 19.8 | 2.8 |
|  |  | 5C | 25702571 | 23940567 | 93.1 | 18619604 | 77.7 | 4675368 | 19.5 | 2.7 |
|  |  | Average | 26386030 | 24573359 | 93.1 | 19107925 | 77.7 | 4777407 | 19.4 | 2.8 |
|  | 700 | 7A | 29807104 | 27910922 | 93.6 | 23588319 | 84.5 | 3616849 | 12.9 | 2.5 |
|  |  | 7B | 26162690 | 24625490 | 94.1 | 20827395 | 84.5 | 3168514 | 12.8 | 2.5 |
|  |  | 7C | 25883732 | 24238764 | 93.6 | 20078961 | 82.8 | 3461021 | 14.2 | 2.8 |
|  |  | Average | 27284508 | 25591725 | 93.8 | 21498225 | 83.9 | 3415461 | 13.3 | 2.7 |
| **Total average** |  |  | 27883515 | 25910839 | 92.9 | 21003684 | 80.9 | 4135873 | 16.1 | 2.9 |
