# Supplementary material for: Transcriptomic Leaf Profiling Reveals Differential Responses of the Two Most Traded Coffee Species to Elevated [CO2]
Source: Int J Mol Sci. 2020 Dec 3;21(23):9211. doi: 10.3390/ijms21239211 (PMC7730880; doi:10.3390/ijms21239211)
Supplement: Supplementary file 1 [file ijms-21-09211-s001.zip › Table S10.docx]

**Table A10.** Gene Set Enrichment Analysis (GSEA) of differentially expressed genes (DEGs), considering the response between CL153 and Icatu genotypes under aCO_2_ and eCO_2_. Significantly enriched Gene Ontology (GO) terms from each category – Biological Process (BP), Molecular Function (MF) and Cellular Component (CC) – and KEGG’s metabolic pathways. Values indicate the number of DEGs annotated with each term and pathway (Counts), normalized enrichment scores (NES), p-value and False Discovery Rate (FDR < 0.05).

| **Database** | | **ID** | **Decription** | **Counts** | **NES** | **p-value** | **FDR** |
| --- | --- | --- | --- | --- | --- | --- | --- |
|  | **~~aCO~~_~~2~~_** | | | | | | |
| ~~WikiPathways~~ | | ~~WP3661~~ | ~~genetic interactions between sugar and hormone signaling~~ | ~~12~~ | ~~1.67~~ | ~~2.15E-02~~ | ~~2.90E-02~~ |
|  | **eCO_2_** | | | | | | |
| GO:MF | | GO:0016679 | oxidoreductase activity, acting on diphenols and related substances as donors | 10 | 1.90 | <0.001 | 3.23E-02 |
| GO:CC | | GO:0042170 | plastid membrane | 47 | -1.86 | <0.001 | 1.11E-02 |
