# Supplementary material for: Transcriptomic Leaf Profiling Reveals Differential Responses of the Two Most Traded Coffee Species to Elevated [CO2]
Source: Int J Mol Sci. 2020 Dec 3;21(23):9211. doi: 10.3390/ijms21239211 (PMC7730880; doi:10.3390/ijms21239211)
Supplement: Supplementary file 1 [file ijms-21-09211-s001.zip › Table S11.docx]

**Table A11.** Icatu and CL153 responsive photosynthetic-related DEGs to the enhancement of [CO2]. The searched GO-terms included: “Photosynthesis”, “Chlorophyll metabolic process”, “Ribulose-bisphosphate carboxylase activity”, “Antioxidant activity”, “Lipid metabolic process (LOX, FAD)”, “Cellular respiration”, “Malate dehydrogenase activity”, and “Pyruvate kinase activity”. Blue indicates up-regulation and yellow indicates down-regulation.

| **GENE** | **PROTEIN** | **FUNCTION** | **ICATU** | | **CL 153** | |
| --- | --- | --- | --- | --- | --- | --- |
|  | **Photosynthesis** | |  | |  | |
| Cc01_g13010 | PGR5-like protein 1B, chloroplastic | Electron transport, Transport | 0.874 | | 0.441 | |
| Cc01_g17770 | Photosystem II reaction center PSB28 chloroplastic | Photosynthesis | 0.455 | | -0.500 | |
| Cc02_g20480 | K(+) efflux antiporter 3, chloroplastic | Antiport,Ion transport,Potassium transport,Transport | 0.948 | | -0.259 | |
| Cc02_g28180 | Ferredoxin-thioredoxin variable chain | Oxidoreductase | 0.406 | | 0.142 | |
| Cc02_g37870 | UPF0187 protein At3g61320, chloroplastic | Photosynthesis | 0.879 | |  | |
| Cc03_g02730 | Pyruvate, phosphate dikinase, chloroplastic | Kinase, Transferase | 2.655 | |  | |
| Cc04_g02050 | Thioredoxin-like 2- chloroplastic | Electron transport,Transport | 0.411 | |  | |
| Cc04_g16560 | Geranylgeranyl diphosphate chloroplastic | Oxidoreductase | 0.150 | |  | |
| Cc05_g04890 | Glucose-6-phosphate phosphate translocator chloroplastic | Sugar transport,Transport | 1.246 | | 1.125 | |
| Cc05_g09930 | Chlorophyll a-b binding protein 8, chloroplastic | Photosynthesis | 0.545 | | 0.129 | |
| Cc05_g12720 | Chlorophyll a-b binding protein 13, chloroplastic | Photosynthesis | 0.620 | |  | |
| Cc06_g08470 | Heme oxygenase 1, chloroplastic | Oxidoreductase | 1.418 | | 1.132 | |
| Cc06_g17100 | Magnesium-chelatase subunit H | Ligase | 0.770 | | 0.384 | |
| Cc06_g22740 | Magnesium-protoporphyrin IX monomethyl ester [oxidative] cyclase, chloroplastic | Oxidoreductase | 0.530 | | 0.195 | |
| Cc06_g23280 | Photosystem II 5 kDa chloroplastic | Photosynthesis | 0.367 | | -0.137 | |
| Cc09_g08490 | Photosystem I reaction center subunit chloroplastic | Photosynthesis | 0.489 | | 0.290 | |
| Cc10_g05650 | Zinc finger ZAT10 | Repressor | 1.306 | | 1.453 | |
| Cc10_g11890 | Photosystem II 22 kDa chloroplastic | Photosynthesis | 0.481 | | -0.559 | |
| Cc10_g12590 | Photosystem I reaction center subunit chloroplastic | Photosynthesis | 0.371 | | 0.416 | |
| Cc10_g12990 | PsbP domain-containing protein 4, chloroplastic | Hydrolase | 0.502 | | -0.148 | |
| Cc11_g10810 | Phosphoenolpyruvate carboxylase 4 | Lyase | 0.528 | | -0.274 | |
| Cc00_g24160 | Dihydrodipicolinate reductase chloroplastic | Chaperone,Oxidoreductase | -0.372 | | -0.816 | |
| Cc01_g06000 | Magnesium-chelatase subunit chloroplastic | Ligase | -0.376 | | -0.843 | |
| Cc01_g15890 | Photosystem I reaction center subunit chloroplastic | Photosynthesis | -0.343 | | -0.295 | |
| Cc02_g11770 | Oxygen-evolving enhancer chloroplastic | Photosynthesis | -0.456 | | -0.437 | |
| Cc02_g27800 | Thylakoid lumenal 19 kDa chloroplastic | Photosynthesis | -0.171 | | -0.335 | |
| Cc11_g04720 | PsbP-like protein 1, chloroplastic | Photosynthesis | -0.292 | | -0.954 | |
| Cc03_g03590 | Photosystem I reaction center subunit chloroplastic | Photosynthesis | -0.265 | | -0.621 | |
| Cc04_g16410 | Chlorophyll a-b binding protein P4 chloroplastic | Photosynthesis | -0.326 | | -0.639 | |
| Cc05_g06850 | Protochlorophyllide chloroplastic | Oxidoreductase | -0.407 | | -0.759 | |
| Cc05_g09650 | Chlorophyll a-b binding chloroplastic (Fragments) | Photosynthesis | -1.623 | | -0.872 | |
| Cc05_g11180 | Phosphoenolpyruvate carboxylase | Allosteric enzyme, Lyase | -0.523 | | -1.508 | |
| Cc06_g01460 | Chlorophyll a-b binding protein 4.1 chloroplastic | Photosynthesis | -0.315 | | -0.665 | |
| Cc06_g20190 | PsbP domain-containing protein 6, chloroplastic | Photosynthesis | -0.197 | | -1.023 | |
| Cc07_g10820 | Ferredoxin--NADP leaf-type chloroplastic | Oxidoreductase | -0.219 | | -0.760 | |
| Cc07_g18500 | Magnesium-chelatase subunit chloroplastic | Ligase | -0.396 | | -0.822 | |
| Cc08_g05240 | Protein PAM68, chloroplastic | Photosynthesis | -0.603 | | -0.374 | |
| Cc08_g08290 | PsbP domain-containing protein 5, chloroplastic | Photosynthesis | -0.765 | | -1.487 | |
| Cc08_g13730 | PGR5-like protein 1A, chloroplastic | Electron transport,Transport | -0.114 | | -0.492 | |
| Cc09_g02010 | Chlorophyll a-b binding chloroplastic | Photosynthesis | -0.542 | | -0.722 | |
| Cc09_g03670 | Isopentenyl-diphosphate Delta-isomerase II | Isomerase | -0.302 | | 1.224 | |
| Cc09_g06610 | Photosystem I reaction center subunit chloroplastic | Photosynthesis | -0.223 | | -0.419 | |
| Cc09_g08000 | Ferredoxin--NADP root chloroplastic | Oxidoreductase | -0.433 | | -0.464 | |
| Cc09_g09010 | Chlorophyll a-b binding protein 21, chloroplastic | Photosynthesis | -0.477 | | -0.389 | |
| Cc09_g09500 | Chlorophyll a-b binding protein 36, chloroplastic | Photosynthesis | -0.546 | | -0.935 | |
| Cc10_g04470 | Probable inactive heme oxygenase chloroplastic | Photosynthesis | -0.582 | | -0.624 | |
| Cc10_g16210 | Chlorophyll a-b binding protein 1, chloroplastic | Photosynthesis | -0.182 | | -0.630 | |
| Cc11_g02280 | Photosystem II stability assembly factor chloroplastic | Photosynthesis | -0.167 | | -1.004 | |
| Cc02_g20330 | Probable pectin methylesterase CGR2 | Methyltransferase,Transferase |  | | 0.729 | |
| Cc00_g03620 | Protein ACTIVITY OF BC1 COMPLEX KINASE 1, chloroplastic | Kinase, Transferase |  | | -0.596 | |
| Cc01_g17500 | Photosystem I reaction center subunit IV chloroplastic | Photosynthesis |  | | -0.271 | |
| Cc02_g08380 | Photosystem II reaction center PSB28 chloroplastic | Photosynthesis |  | | -0.832 | |
| Cc02_g37680 | K(+) efflux antiporter chloroplastic | Antiport, Ion transport,Potassium transport,Transport |  | | -0.415 | |
| Cc02_g33910 | Ferredoxin-thioredoxin variable chain | Oxidoreductase |  | | -0.965 | |
| Cc04_g03050 | Photosystem I reaction center subunit chloroplastic | Photosynthesis |  | | -0.266 | |
| Cc02_g23630 | 2-C-methyl-D-erythritol 4-phosphate chloroplastic | Kinase, Transferase |  | | -0.455 | |
| Cc02_g11050 | Thioredoxin-like 2- chloroplastic | Electron transport,Transport |  | | -0.703 | |
| Cc10_g03310 | Geranylgeranyl diphosphate chloroplastic | Oxidoreductase |  | | -0.446 | |
| Cc11_g17260 | Glucose-6-phosphate phosphate translocator chloroplastic | Sugar transport,Transport |  | | -0.202 | |
| Cc10_g00140 | Chlorophyll a-b binding chloroplastic | Photosynthesis |  | | -0.400 | |
| Cc11_g16910 | Chlorophyll a-b binding chloroplastic | Photosynthesis |  | | -0.457 | |
| Cc11_g16230 | Photosystem II core complex s chloroplastic | Oxidoreductase |  | | -0.065 | |
| Cc03_g13110 | Tetrapyrrole-binding chloroplastic | Photosynthesis | 0.667 | | 0.681 | |
| Cc08_g02980 | Zinc finger ZAT11 | Repressor |  | | -0.438 | |
| Cc11_g11320 | Protein PHYLLO, chloroplastic | Photosynthesis |  | | -0.845 | |
| Cc10_g12660 | Protein translocase subunit SECA2, chloroplastic | Photosynthesis | -0.527 | | -1.702 | |
| Cc07_g05350 | Oxygen-evolving enhancer chloroplastic | Hydrolase | -0.187 | | -0.604 | |
| Cc06_g00310 | Serine threonine- kinase chloroplastic | Lyase | 0.757 | | -0.346 | |
| Cc03_g06030 | PROTON GRADIENT REGULATION chloroplastic | Chaperone,Oxidoreductase | 2.535 | | 1.115 | |
| Cc02_g06960 | Sedoheptulose-1,7-bisphosphatase, chloroplastic | Ligase | 0.523 | | -0.504 | |
| Cc06_g01860 | Fructose-1,6-bisphosphatase, chloroplastic | Photosynthesis | 0.315 | |  | |
| Cc02_g21540 | Copper-transporting ATPase chloroplastic | Photosynthesis | -0.416 | |  | |
| Cc06_g00890 | Ferritin-1, chloroplastic | Photosynthesis | -0.856 | | -0.371 | |
| Cc05_g01540 | ATP-dependent zinc metalloprotease FTSH chloroplastic | Photosynthesis |  | | -1.276 | |
| Cc02_g18430 | Pentatricopeptide repeat-containing chloroplastic | Photosynthesis |  | | -0.884 | |
| Cc08_g03960 | Phosphoribulokinase, chloroplastic | Photosynthesis | 0.445 | |  | |
| Cc10_g01790 | Protein disulfide-isomerase LQY1, chloroplastic | Oxidoreductase | -0.348 | | -0.613 | |
| Cc02_g21250 | 2-carboxy-1,4-naphthoquinone phytyltransferase, chloroplastic | Photosynthesis | -0.216 | | -0.593 | |
| Cc02_g16540 | Triosephosphate chloroplastic | Allosteric enzyme, Lyase |  | | -0.205 | |
| Cc07_g03310 | Protease Do-like chloroplastic | Photosynthesis | -0.375 | | -0.689 | |
| Cc04_g04380 | Palmitoyl-monogalactosyldiacylglycerol delta-7 chloroplastic | Photosynthesis | -1.210 | | -0.770 | |
| Cc07_g08150 | Protease Do-like 7 | Oxidoreductase |  | | -0.130 | |
| Cc02_g24950 | Peptidyl-prolyl cis-trans isomerase FKBP20-2, chloroplastic | Ligase |  | | -0.800 | |
| Cc10_g00410 | Glyceraldehyde-3-phosphate dehydrogenase chloroplastic | Photosynthesis | 0.791 | |  | |
| Cc11_g00610 | Glyceraldehyde-3-phosphate dehydrogenase chloroplastic (Fragment) | Electron transport,Transport | 0.657 | | -0.128 | |
| Cc07_g09720 | Phosphopantetheine adenylyltransferase | Photosynthesis | -0.449 | | -0.397 | |
| Cc04_g15130 | Ribulose-phosphate 3- chloroplastic (Fragment) | Isomerase | 0.118 | | -0.291 | |
| Cc07_g02160 | Carbonic anhydrase, chloroplastic | Photosynthesis |  | | -0.666 | |
| Cc05_g08640 | ATP-dependent zinc metalloprotease FTSH chloroplastic | Oxidoreductase | 0.508 | | -0.511 | |
| Cc06_g00240 | Chlorophyll a-b binding protein 51, chloroplastic | Photosynthesis |  | | -0.596 | |
| Cc02_g01340 | Chlorophyll a-b binding protein 66, chloroplastic | Photosynthesis | 1.542 | | 0.533 | |
| Cc05_g10510 | Solanesyl diphosphate synthase 1 | Photosynthesis | 1.951 | | 0.507 | |
| Cc07_g12510 | ATP-dependent zinc metalloprotease FTSH chloroplastic | Photosynthesis | 5.195 | | 0.885 | |
| Cc06_g08290 | Thylakoid lumenal kDa chloroplastic | Methyltransferase,Transferase | -0.633 | | -0.868 | |
| Cc10_g00010 | ACTIVITY OF BC1 COMPLEX KINASE chloroplastic | Kinase, Transferase | 0.996 | | 0.982 | |
| Cc10_g00150 | Calvin cycle CP12- chloroplastic | Photosynthesis | 0.327 | |  | |
| Cc04_g02720 | ATP-dependent zinc metalloprotease 3 | Photosynthesis | -0.392 | | -0.853 | |
|  | **Chlorophyll metabolic process** | | |  | |  |
| Cc04_g03230 | Pyridoxal 5 -phosphate synthase subunit | Lyase | 0.570 | | 0.365 | |
| Cc04_g16560 | Geranylgeranyl diphosphate chloroplastic | Oxidoreductase | 0.150 | |  | |
| Cc06_g22740 | Magnesium-protoporphyrin IX monomethyl este | Oxidoreductase | 0.530 | | 0.195 | |
| Cc07_g00790 | Sulfite oxidase | Oxidoreductase | 1.013 | | 0.364 | |
| Cc08_g09240 | Tocopherol chloroplastic | Isomerase | 0.337 | | -0.260 | |
| Cc09_g03200 | Violaxanthin de-epoxidase, chloroplastic | Oxidoreductase | 0.450 | | -0.178 | |
| Cc10_g12990 | PsbP domain-containing protein 4, chloroplastic | Hydrolase | 0.502 | | -0.148 | |
| Cc01_g06000 | Magnesium-chelatase subunit chloroplastic | Ligase | -0.376 | | -0.843 | |
| Cc05_g06850 | Protochlorophyllide chloroplastic | Oxidoreductase | -0.407 | | -0.759 | |
| Cc07_g18500 | Magnesium-chelatase subunit chloroplastic | Ligase | -0.396 | | -0.822 | |
| Cc09_g03670 | Isopentenyl-diphosphate Delta-isomerase II | Isomerase | -0.302 | | 1.224 | |
| Cc00_g03620 | Protein ACTIVITY OF BC1 COMPLEX KINASE chloroplastic | Kinase, Transferase |  | | -0.596 | |
| Cc10_g08170 | Glutamyl-tRNA reductase 1, chloroplastic | Oxidoreductase |  | | 0.246 | |
| Cc10_g12640 | Glutamate-1-semialdehyde 2,1-aminomutase, chloroplastic | Isomerase | -0.567 | | -1.242 | |
| Cc09_g04450 | Oxygen-dependent coproporphyrinogen-III oxidase, chloroplastic | Oxidoreductase | -0.310 | | -0.719 | |
| Cc06_g17670 | NADPH-dependent thioredoxin reductase 3 | Oxidoreductase |  | | -0.712 | |
| Cc06_g17810 | Zeatin O-glucosyltransferase | Glycosyltransferase, Transferase | 2.145 | |  | |
| Cc01_g14350 | U-box domain-containing protein 44 | Transferase | 0.309 | | 0.299 | |
| Cc07_g04870 | Protoporphyrinogen oxidase 2 | Oxidoreductase | -1.778 | | -0.841 | |
| Cc08_g06800 | Delta-aminolevulinic acid dehydratase, chloroplastic | Allosteric enzyme, Lyase | -0.266 | | -0.732 | |
| Cc01_g19080 | Chlorophyllase-1 | Hydrolase |  | | 0.182 | |
| Cc02_g05570 | Uroporphyrinogen decarboxylase, chloroplastic | Decarboxylase, Lyase | -0.653 | | -0.661 | |
| Cc02_g32140 | Chlorophyll(ide) b reductase NOL, chloroplastic | Oxidoreductase |  | | -0.751 | |
| Cc11_g17030 | Histidine kinase 3 | Developmental protein, Kinase, Transferase |  | | -0.271 | |
| Cc06_g11380 | Putative GATA transcription factor 22 | DNA-binding | 1.475 | | 0.560 | |
| Cc03_g05380 | Electron transfer flavoprotein subunit beta, mitochondrial | Electron transport, Transport | 0.320 | |  | |
| Cc11_g01700 | 1-deoxy-D-xylulose-5-phosphate synthase 1, chloroplastic | Transferase | -0.326 | | -0.480 | |
| Cc06_g09730 | Probable chlorophyll(ide) b reductase NYC1, chloroplastic | Oxidoreductase | -0.161 | |  | |
| Cc05_g02360 | Histidine kinase 2 | Protein binding | -0.618 | | -0.688 | |
| Cc03_g04230 | Chlorophyllase-2, chloroplastic | Hydrolase | -1.201 | | -0.805 | |
| Cc06_g01120 | Chlorophyll synthase, chloroplastic | Transferase |  | | -0.342 | |
| Cc01_g10220 | Pheophorbide a oxygenase, chloroplastic | Oxidoreductase | 0.255 | | -0.465 | |
| Cc10_g11980 | Chlorophyllide a oxygenase, chloroplastic | Oxidoreductase | 2.516 | | 1.044 | |
| Cc03_g11370 | Red chlorophyll catabolite reductase, chloroplastic | Oxidoreductase | -0.315 | | -0.517 | |
| Cc05_g11120 | Uroporphyrinogen decarboxylase 1, chloroplastic | Decarboxylase, Lyase | -0.444 | | -0.883 | |
| Cc11_g16330 | Two-component response regulator ARR1 | Activator, DNA-binding | 0.304 | | 0.261 | |
| Cc05_g16090 | Porphobilinogen deaminase, chloroplastic | Transferase | -0.650 | | -0.438 | |
| Cc01_g21760 | Protoporphyrinogen oxidase, chloroplastic | Oxidoreductase | -0.345 | | -0.469 | |
| Cc01_g15510 | ABC transporter B family member 25, mitochondrial | Ion transport, Iron transport, Transport |  | | -0.375 | |
| Cc01_g17430 | Transcription factor PIF1 | Activator, DNA-binding | 0.765 | | 0.900 | |
| Cc10_g03990 | Uroporphyrinogen-III synthase, chloroplastic | Lyase | -0.760 | | -2.141 | |
|  | **Ribulose-bisphosphate carboxylase activity** | | | | |  |
| Cc00_g15710 | Ribulose bisphosphate carboxylase small chain chloroplastic | Lyase, Monooxygenase, Oxidoreductase | -0.291 | | -0.457 | |
| Cc02_g07500 | Ribulose bisphosphate carboxylase/oxygenase activase 1, chloroplastic | Lyase,Monooxygenase,Oxidoreductase | 1.024 | | 0.078 | |
| Cc01_g10720 | RuBisCO large subunit-binding protein subunit beta, chloroplastic | Chaperone | -0.348 | | -1.415 | |
|  | **Antioxidant activity** | |  | |  | |
| Cc07_g20150 | Lysine-specific demethylase 3B | Dioxygenase, Oxidoreductase | -0.259 | |  | |
| Cc11_g11380 | Peroxiredoxin-like 2A | Antioxidant |  | | 2.389 | |
| Cc07_g12520 | Lysine-specific demethylase 3B | Dioxygenase, Oxidoreductase |  | | -0.377 | |
| Cc07_g11710 | Catalase isozyme 1 | Oxidoreductase, Peroxidase | 1.672 | | -0.313 | |
| Cc08_g14620 | Probable glutathione peroxidase 8 | Oxidoreductase, Peroxidase | -0.325 | |  | |
| Cc08_g07250 | Phospholipid hydroperoxide glutathione peroxidase, chloroplastic | Oxidoreductase, Peroxidase | 0.256 | | -0.228 | |
| Cc06_g07850 | Bifunctional purple acid phosphatase 26 | Hydrolase, Oxidoreductase, Peroxidase | 0.456 | | 0.774 | |
| Cc10_g12080 | L-ascorbate peroxidase T, chloroplastic | Oxidoreductase, Peroxidase |  | | -0.214 | |
| Cc08_g15360 | Respiratory burst oxidase homolog protein A | Oxidoreductase, Peroxidase | 0.768 | | 2.155 | |
| Cc07_g03760 | Chorismate mutase 1, chloroplastic | Allosteric enzyme, Isomerase | 0.585 | | 1.456 | |
| Cc00_g17550 | Peroxidase 3 | Oxidoreductase, Peroxidase | -1.373 | | 1.917 | |
| Cc01_g15110 | Peroxidase 4 | Oxidoreductase, Peroxidase | -1.993 | |  | |
| Cc07_g17340 | Peroxidase 5 | Oxidoreductase, Peroxidase | -1.852 | |  | |
| Cc02_g18480 | Peroxidase 10 | Oxidoreductase, Peroxidase | -1.788 | | -1.092 | |
| Cc11_g11190 | Peroxidase 11 | Oxidoreductase, Peroxidase |  | | 3.611 | |
| Cc10_g15150 | Peroxidase 12 | Oxidoreductase, Peroxidase |  | | 1.568 | |
| Cc06_g13090 | Peroxidase 16 | Oxidoreductase, Peroxidase | -0.542 | | 0.202 | |
| Cc06_g08460 | Peroxidase 25 | Oxidoreductase, Peroxidase | 2.246 | |  | |
| Cc09_g01700 | Peroxidase 42 | Oxidoreductase, Peroxidase |  | | 1.764 | |
| Cc02_g21960 | Peroxidase 43 | Oxidoreductase, Peroxidase | 0.475 | | 2.094 | |
| Cc00_g00940 | Peroxidase 47 | Oxidoreductase, Peroxidase |  | | 1.351 | |
| Cc01_g08210 | Peroxidase 64 | Oxidoreductase, Peroxidase | -0.646 | | -2.155 | |
| Cc02_g03550 | Peroxidase 66 | Oxidoreductase, Peroxidase | -0.417 | |  | |
| Cc07_g10080 | Peroxidase 72 | Oxidoreductase, Peroxidase |  | | 2.813 | |
| Cc07_g06870 | Peroxidase 73 | Oxidoreductase, Peroxidase | -1.567 | | 0.601 | |
| Cc06_g03490 | L-ascorbate peroxidase 2 | Oxidoreductase, Peroxidase |  | | 1.455 | |
| Cc02_g16260 | L-ascorbate peroxidase 3 | Oxidoreductase, Peroxidase | 0.304 | | -0.333 | |
| Cc08_g00640 | Putative L-ascorbate peroxidase 6 | Oxidoreductase, Peroxidase |  | | -0.734 | |
| Cc08_g14600 | Probable phospholipid hydroperoxide glutathione peroxidase | Oxidoreductase, Peroxidase | 0.941 | | 0.221 | |
| Cc06_g06390 | 2-Cys peroxiredoxin BAS1-like, chloroplastic | Antioxidant, Oxidoreductase, Peroxidase |  | | -0.393 | |
| Cc04_g09270 | Purple acid phosphatase 17 | Hydrolase, Oxidoreductase | 0.806 | | 1.512 | |
| Cc00_g03790 | Probable glutathione peroxidase 2 | Oxidoreductase, Peroxidase | 0.221 | | -0.138 | |
| Cc01_g19740 | Probable glutathione peroxidase 4 | Oxidoreductase, Peroxidase | 0.456 | | 0.339 | |
| Cc07_g01940 | Glutaredoxin-C2 | Electron transport, Transport | 0.639 | | 0.251 | |
| Cc06_g12910 | Glutaredoxin-C4 | Electron transport, Transport |  | | 0.377 | |
| Cc02_g08510 | Glutathione S-transferase DHAR2 | Oxidoreductase, Transferase | 0.309 | | 0.969 | |
| Cc10_g06730 | Thylakoid lumenal 29 kDa protein, chloroplastic | Oxidoreductase | -0.247 | | -0.491 | |
| Cc06_g12630 | Respiratory burst oxidase homolog protein E | Oxidoreductase, Peroxidase | -1.025 | |  | |
| Cc10_g06720 | Thylakoid lumenal 29 kDa protein, chloroplastic | Oxidoreductase | -0.243 | | -0.863 | |
| Cc07_g13580 | Glutathione S-transferase DHAR3, chloroplastic | Oxidoreductase, Transferase | 0.514 | | -0.427 | |
| Cc01_g11800 | L-ascorbate peroxidase 1, cytosolic | Oxidoreductase, Peroxidase | -0.429 | | -1.010 | |
| Cc06_g05140 | Peroxiredoxin-2E, chloroplastic | Antioxidant, Oxidoreductase, Peroxidase | -0.226 | | -1.002 | |
|  | **Lipid metabolic process** | | |  | |  |
| Cc02_g33780 | Probable linoleate 9S-lipoxygenase 5 | Dioxygenase, Oxidoreductase | 0.287 | |  | |
| Cc01_g04060 | Linoleate 13S-lipoxygenase 2-1, chloroplastic | Dioxygenase, Oxidoreductase | -2.515 | | -2.350 | |
| Cc02_g13400 | Lipoxygenase 3, chloroplastic | Dioxygenase, Oxidoreductase | 0.785 | |  | |
| Cc00_g30760 | Linoleate 13S-lipoxygenase 3-1, chloroplastic | Dioxygenase, Oxidoreductase | -1.637 | | -2.379 | |
| Cc03_g03580 | Linoleate 9S-lipoxygenase 5 | Dioxygenase, Oxidoreductase | -0.627 | | 0.292 | |
| Cc11_g16680 | Lipoxygenase 6, chloroplastic | Dioxygenase, Oxidoreductase | 0.255 | | 0.288 | |
| Cc01_g05180 | Omega-6 fatty acid desaturase, endoplasmic reticulum isozyme 2 | Oxidoreductase | 0.506 | | 0.666 | |
| Cc02_g06400 | Omega-3 fatty acid desaturase, chloroplastic | Oxidoreductase | 0.585 | | 0.427 | |
|  | **Cellular respiration** | |  | |  | |
| Cc02_g20400 | Malate glyoxysomal | Transferase | 1.203 | | 0.403 | |
| Cc03_g05530 | Malate cytoplasmic | Oxidoreductase | 0.362 | | 0.561 | |
| Cc11_g10810 | Phosphoenolpyruvate carboxylase 4 | Lyase | 0.528 | | -0.274 | |
| Cc05_g11180 | Phosphoenolpyruvate carboxylase | Allosteric enzyme, Lyase | -0.523 | | -1.508 | |
| Cc07_g01320 | Cytochrome b-c1 complex subunit 9 | Electron transport, Respiratory chain, Transport | 0.637 | | 0.606 | |
| Cc08_g05330 | Ubiquinol oxidase 3, mitocondrial | Oxidoreductase | -0.485 | | -1.159 | |
| Cc06_g04000 | Isocitrate dehydrogenase [NAD] catalytic subunit 5, mitocondrial | Oxidoreductase | 0.200 | | 0.426 | |
| Cc07_g09450 | Succinate dehydrogenase [ubiquinone] flavoprotein subunit 1, mitochondrial | Oxidoreductase | 0.406 | |  | |
| Cc08_g07680 | Kelch repeat-containing protein At3g27220 | Transmenbrane |  | | -0.627 | |
| Cc08_g09040 | Aspartate aminotransferase, cytoplasmic | Aminotransferase, Transferase | 0.638 | | 0.578 | |
| Cc04_g00910 | Probable complex I intermediate-associated protein 30 | Chaperone | 0.329 | | -0.338 | |
| Cc06_g20970 | Succinate dehydrogenase [ubiquinone] iron-sulfur subunit 2, mitochondrial | Oxidoreductase | 0.899 | | 0.488 | |
| Cc04_g03950 | Cytochrome c oxidase subunit 5b-1, mitochondrial | Metal-binding, Zinc |  | | 0.337 | |
| Cc05_g09160 | Citrate synthase, glyoxysomal | Transferase | 0.252 | |  | |
| Cc07_g12860 | Isocitrate dehydrogenase [NADP] | Oxidoreductase | -0.117 | | 0.193 | |
| Cc05_g08360 | Phosphoenolpyruvate carboxylase | Allosteric enzyme, Lyase | 0.792 | | 0.340 | |
| Cc07_g10140 | D-2-hydroxyglutarate dehydrogenase, mitochondrial | Oxidoreductase |  | | -0.542 | |
| Cc11_g01730 | Cytochrome b-c1 complex subunit 7 | Electron transport, Respiratory chain, Transport | 0.352 | |  | |
| Cc07_g07610 | Cytochrome c oxidase subunit 6a, mitochondrial | Oxidase | 0.191 | | 0.545 | |
| Cc02_g23400 | Cytochrome c | Electron transport, Respiratory chain, Transport |  | | 0.483 | |
| Cc09_g10650 | Isocitrate dehydrogenase [NAD] regulatory subunit 1, mitochondrial | Oxidoreductase | 0.342 | | 0.682 | |
| Cc02_g02230 | Probable NADH dehydrogenase [ubiquinone] 1 alpha subcomplex subunit 5, mitochondrial | Oxidoreductase |  | | 0.205 | |
|  | **Malate dehydrogenase activity** | | |  | |  |
| Cc02_g20400 | Malate glyoxysomal | Transferase | 1.203 | | 0.403 | |
| Cc03_g05530 | Malate cytoplasmic | Oxidoreductase | 0.362 | | 0.561 | |
| Cc02_g24520 | Malate cytoplasmic | Oxidoreductase |  | | 1.398 | |
| Cc04_g16570 | Malate dehydrogenase mitochondrial | Oxidoreductase |  | | 0.234 | |
| Cc11_g03270 | 3-isopropylmalate dehydrogenase 2 chloroplastic | Oxidoreductase |  | | -0.340 | |
| Cc06_g10370 | NADP-dependent malic enzyme | Oxidoreductase | -1.161 | |  | |
| Cc04_g10080 | Malate dehydrogenase, glyoxysomal | Oxidoreductase | -1.046 | | 0.603 | |
|  | **Pyruvate kinase activity** | | |  | |  |
| Cc01_g00290 | Pyruvate cytosolic isozyme | Kinase, Transferase | 0.208 | | -0.183 | |
| Cc03_g04490 | Pyruvate kinase isozyme chloroplastic | Kinase,Transferase | 0.318 | | 0.165 | |
| Cc02_g02100 | Plastidial pyruvate kinase 2 | Kinase,Transferase |  | | 0.507 | |
