# Supplementary material for: Transcriptomic Leaf Profiling Reveals Differential Responses of the Two Most Traded Coffee Species to Elevated [CO2]
Source: Int J Mol Sci. 2020 Dec 3;21(23):9211. doi: 10.3390/ijms21239211 (PMC7730880; doi:10.3390/ijms21239211)
Supplement: Supplementary file 1 [file ijms-21-09211-s001.zip › Table S2.docx]

**Table A2.** Total gene expression and differentially expressed genes (DEGs) counts of Icatu and CL153 samples, grown at aCO_2_ and aCO_2_. Genotype: Number of total expressed genes at aCO_2_ and at eCO_2_ in Icatu and CL153 genotypes, number of all, unknown and annotated DEGs at eCO_2_ vs. aCO_2_, with the respective number of up- and down-regulated DEGs. [CO2]: Number of total expressed genes by Icatu and by CL153 in aCO_2_ and eCO_2_, number of all, unknown and annotated and annotated DEGs in CL153 vs. Icatu, with the respective number of up- and down-regulated DEGs.

|  | **Expressed Genes** | |  | **DEGs** | | | | | | | | | | |
| --- | --- | --- | --- | --- | --- | --- | --- | --- | --- | --- | --- | --- | --- | --- |
|  | Control | Treatment |  | All | | |  | Unknown | | |  | Annotated | | |
| **Genotype** | **aCO_2_** | **eCO_2_** |  | **Up** | **Down** | **Total** |  | **Up** | **Down** | **Total** |  | **Up** | **Down** | **Total** |
|  |  |  |  |  |  |  |  |  |  |  |  |  |  |  |
| Icatu | 21714 | 21659 |  | 3357 | 3073 | 6430 |  | 822 | 713 | 1535 |  | 2535 | 2360 | 4895 |
| CL153 | 20728 | 21186 |  | 4338 | 4386 | 8724 |  | 1184 | 1054 | 2238 |  | 3154 | 3332 | 6486 |
|  |  |  |  |  |  |  |  |  |  |  |  |  |  |  |
| **[CO2]** | **Icatu** | **CL153** |  |  |  |  |  |  |  |  |  |  |  |  |
|  |  |  |  |  |  |  |  |  |  |  |  |  |  |  |
| aCO_2_ | 21714 | 20728 |  | 4118 | 4172 | 8290 |  | 1103 | 1273 | 2376 |  | 3015 | 2899 | 5914 |
| eCO_2_ | 21659 | 21186 |  | 4713 | 4713 | 9426 |  | 1300 | 1362 | 2662 |  | 3413 | 3351 | 6764 |
|  |  |  |  |  |  |  |  |  |  |  |  |  |  |  |
