# Supplementary material for: Transcriptomic Leaf Profiling Reveals Differential Responses of the Two Most Traded Coffee Species to Elevated [CO2]
Source: Int J Mol Sci. 2020 Dec 3;21(23):9211. doi: 10.3390/ijms21239211 (PMC7730880; doi:10.3390/ijms21239211)
Supplement: Supplementary file 1 [file ijms-21-09211-s001.zip › Table S9.docx]

**Table A9.** Gene Set Enrichment Analysis (GSEA) of differentially expressed genes (DEGs), considering the effect of eCO_2_ in Icatu and CL153, performed with WEB-based Gene SeT AnaLysis Toolkit (WebGestalt). Significantly enriched Gene Ontology (GO) terms from each category – Biological Process (BP), Molecular Function (MF) and Cellular Component (CC) – and KEGG’s metabolic pathways. Values indicate the number of DEGs annotated with each term and pathway (Counts), normalized enrichment scores (NES), p-value and False Discovery Rate (FDR < 0.05).

| **Database** | **ID** | | **Decription** | **Counts** | **NES** | **p-value** | **FDR** |
| --- | --- | --- | --- | --- | --- | --- | --- |
|  | | **Icatu** | | | | | |
| GO:BP | GO:0019748 | | secondary metabolic process | 36 | 2.04 | <0.001 | 1.08E-02 |
|  | GO:0009657 | | plastid organization | 31 | 2.01 | <0.001 | 9.59E-03 |
|  | GO:0009642 | | response to light intensity | 18 | 1.91 | <0.001 | 3.22E-02 |
|  | | **CL153** | | | | | |
| GO:BP | GO:0042546 | | cell wall biogenesis | 44 | 2.12 | <0.001 | 7.90E-04 |
|  | GO:0019748 | | secondary metabolic process | 49 | 1.98 | <0.001 | 5.53E-03 |
|  | GO:0044036 | | cell wall macromolecule metabolic process | 27 | 1.97 | <0.001 | 5.27E-03 |
|  | GO:0071669 | | plant-type cell wall organization or biogenesis | 36 | 1.87 | <0.001 | 2.13E-02 |
|  | GO:0005976 | | polysaccharide metabolic process | 65 | 1.81 | <0.001 | 4.71E-02 |
|  | GO:0048646 | | anatomical structure formation involved in morphogenesis | 30 | 1.81 | 1.2E-03 | 4.12E-02 |
|  | GO:0009451 | | RNA modification | 11 | -2.16 | <0.001 | 1.43E-02 |
|  | GO:0034660 | | ncRNA metabolic process | 26 | -2.27 | <0.001 | 6.75E-03 |
| GO:MF | GO:0016798 | | hydrolase activity, acting on glycosyl bonds | 46 | 1.89 | <0.001 | 1.71E-02 |
|  | GO:0016679 | | oxidoreductase activity, acting on diphenols and related substances as donors | 9 | 1.85 | <0.001 | 1.86E-02 |
|  | GO:0016701 | | oxidoreductase activity, acting on single donors with incorporation of molecular oxygen | 7 | 1.78 | <0.001 | 4.55E-02 |
|  | GO:0016758 | | transferase activity, transferring hexosyl groups | 65 | 1.75 | <0.001 | 4.68E-02 |
| GO:CC | GO:0009579 | | thylakoid | 46 | -2.06 | <0.001 | 1.79E-02 |
| ~~KEGG~~ | ~~map00906~~ | | ~~carotenoid biosynthesis~~ | ~~12~~ | ~~1.99~~ | ~~<0.001~~ | ~~8.12E-04~~ |
|  | ~~map00073~~ | | ~~cutin, suberine and wax biosynthesis~~ | ~~5~~ | ~~1.76~~ | ~~1.69E-03~~ | ~~4.91E-02~~ |
