# Supplementary figures and images for: Transcriptomic Leaf Profiling Reveals Differential Responses of the Two Most Traded Coffee Species to Elevated [CO2]
Source: Int J Mol Sci. 2020 Dec 3;21(23):9211. doi: 10.3390/ijms21239211 (PMC7730880; doi:10.3390/ijms21239211)

**A**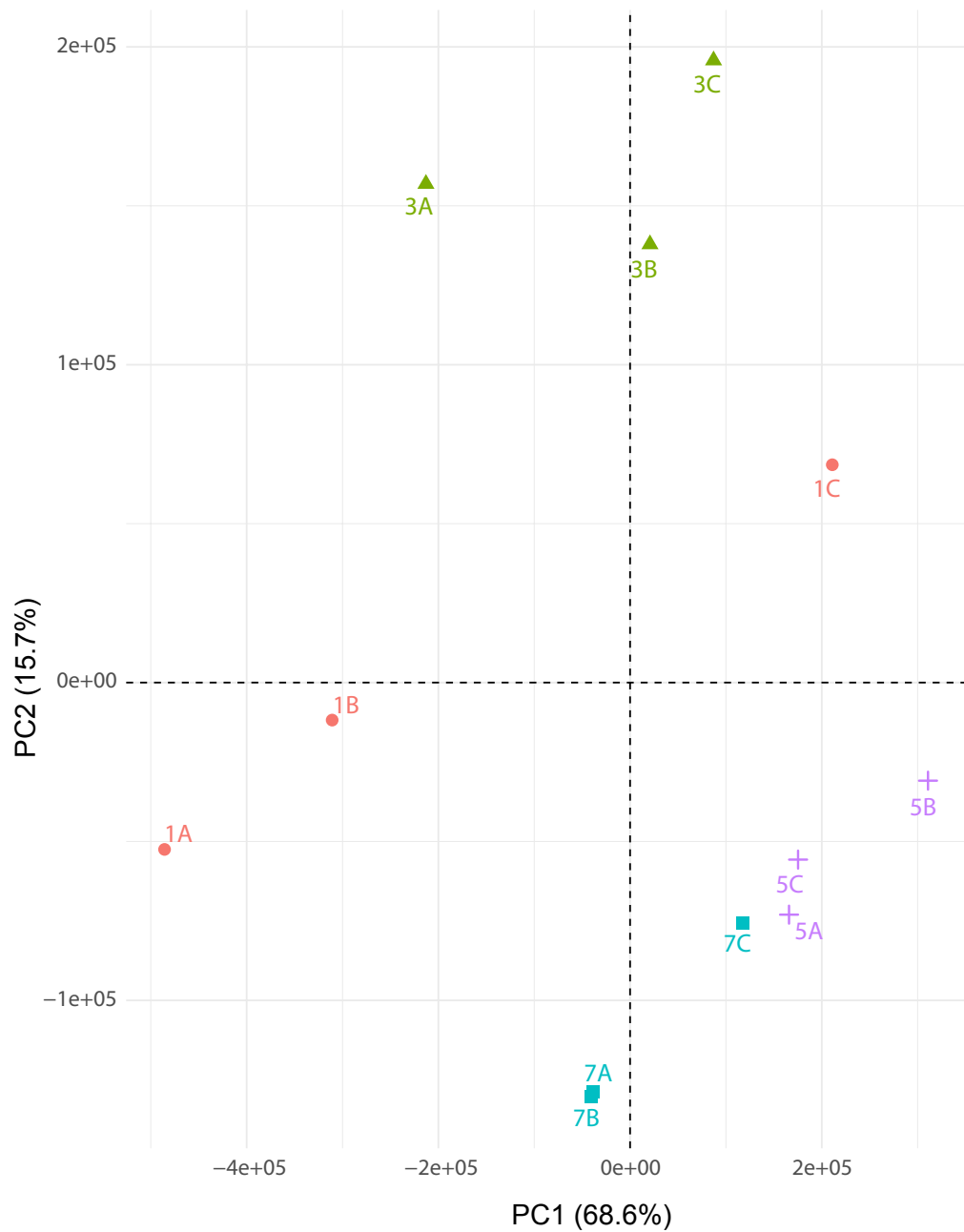**B**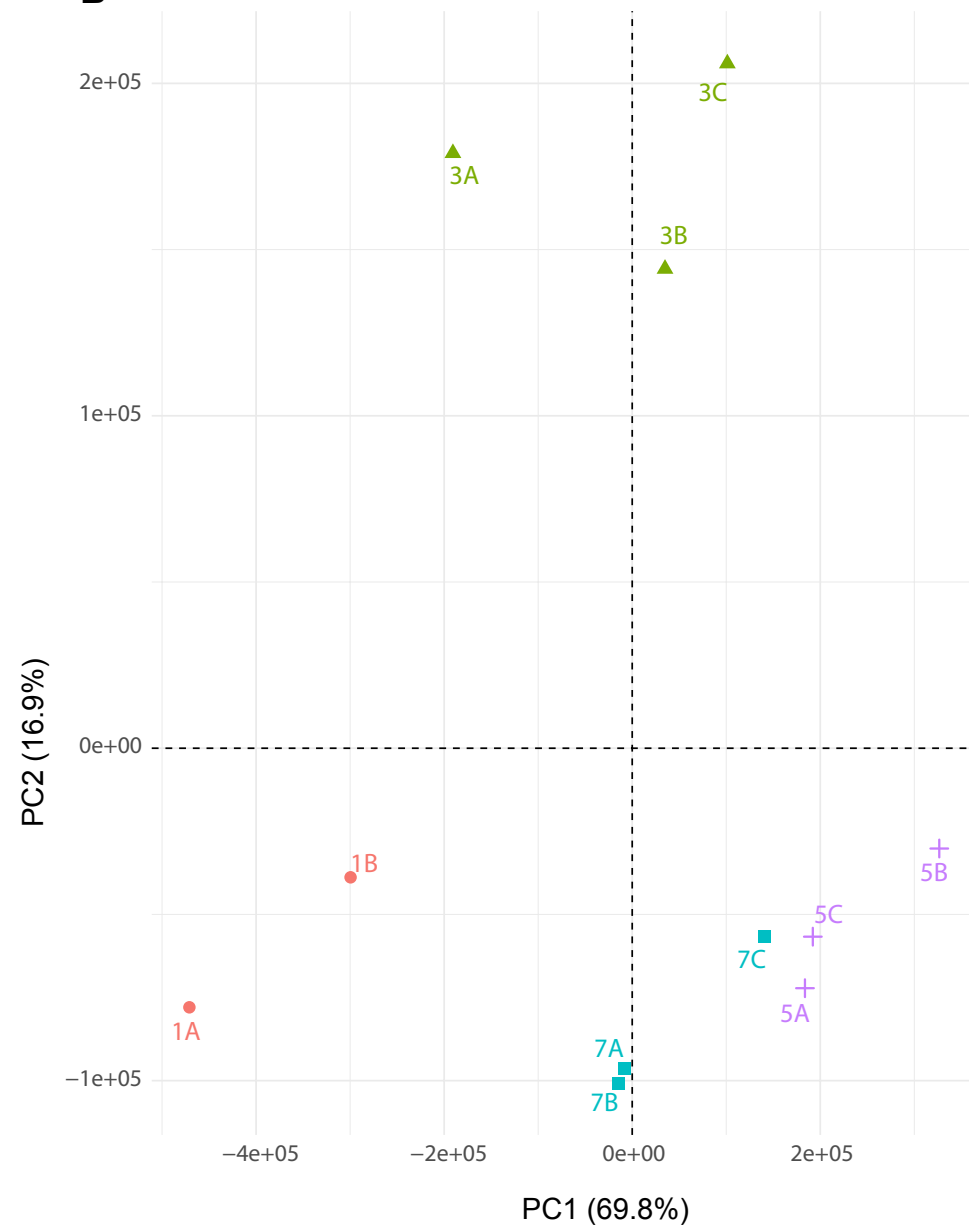

Supplement: Supplementary file 1 [file ijms-21-09211-s001.zip › Figure S1.pdf]
